# Neuroprotection of Truncated Peptide IIAVE from *Isochrysis zhanjiangensis*: Quantum Chemical, Molecular Docking, and Bioactivity Studies

**DOI:** 10.3390/molecules29030692

**Published:** 2024-02-02

**Authors:** Qiuqi Liu, Liyuan Lin, Huijuan Li, Zhong-Ji Qian

**Affiliations:** School of Chemistry and Environment, Guangdong Ocean University, Zhanjiang 524088, China; 15382655907@163.com (Q.L.); liyuanlin1024@163.com (L.L.); juanjie2022@163.com (H.L.)

**Keywords:** *Isochrysis zhanjiangensis*, quantum chemical, molecular docking, SH-SY5Y cells, 6-OHDA, oxidative stress, neuroprotection

## Abstract

Parkinson’s disease (PD) is a progressive neurodegenerative disorder of the elderly for which there is no cure or disease-modifying therapy. Mitochondrial dysfunction and oxidative stress play a central role in dopaminergic neurodegeneration in PD. Therefore, antioxidants are considered a promising neuroprotective approach. In in vivo activity studies, 6-OHDA-induced oxidative stress in SH-SY5Y cells was established as a model of PD for cellular experiments. IIAVE (Ile–Ile–Ala–Val–Glu) was derived from *Isochrysis zhanjiangensis* octapeptide (IIAVEAGC), which has a small molecular weight. The structure and antioxidant activity of IIAVE were tested in a previous study and proved to have good antioxidant potential. In this study, the chemical properties of IIAVE were calculated using quantum chemical methods, including frontier molecular orbital (FMO), molecular electrostatic potential (MEP), natural population analysis (NPA), and global reactivity properties. The interaction of IIAVE with Bcl-2 and DJ-1 was investigated using the molecular docking method. The results showed that IIAVE promoted the activation of the Keap1/Nrf2 pathway and up-regulated the expression of the superoxide dismutase 1 (SOD-1) protein by inhibiting the level of reactive oxygen species (ROS) in cells. In addition, IIAVE inhibits ROS production and prevents 6-OHDA-induced oxidative damage by restoring mitochondrial membrane potential. Furthermore, IIAVE inhibited cell apoptosis by increasing the Bcl-2/Bax ratio and inhibiting the activation of Caspase-9 and Caspase-3. Thus, IIAVE may become a potential drug for the treatment and prevention of PD.

## 1. Introduction

Parkinson’s disease (PD) is the second-most common degenerative disease of the central nervous system. It is characterized by a slow and progressive loss of dopamine (DA) neurons in the substantia nigra (SN) of the midbrain and the accumulation of alpha-synuclein in Lewy bodies and neuritis [1]. Although the etiology of PD is unknown, oxidative stress, lipid peroxidation, and mitochondrial dysfunction are commonly thought to play an important role [2]. Oxidative stress is associated with neurodegenerative processes in the development of Parkinson’s disease. Oxidative stress is caused by overproduction of ROS or defective ROS removal. It can damage cellular lipids, proteins, and DNA. Mitochondrial function itself is affected by oxidative stress [3,4,5], leading to a vicious cycle of further ROS accumulation and mitochondrial damage [6]. This feed-forward mechanism is a common cause of neuronal death in neurodegenerative diseases. Patients with PD have increased superoxide dismutase 1 (SOD-1) activity in the SN region of brain samples, increased oxidative damage to lipids, proteins, and DNA, and reduced mitochondrial activity [7]. Oxidative stress is closely linked to mitochondrial function, and mitochondrial dysfunction is thought to be a component of PD [8]. Mitochondria produce approximately 90% of cellular reactive oxygen species (ROS) [9]. 6-hydroxydopamine (6-OHDA) is a neurotoxin that is widely used in PD models [10]. 6-OHDA induces dopaminergic denaturation dependent on the high affinity of dopaminergic transporter proteins [11]. 6-OHDA enters neuronal cells, crosses the mitochondrial membrane, and inhibits complex I activity in the electron transport chain [12]. The inhibition of complex I increases the production of ROS, which can form toxic hydroxyl radicals or react with nitric oxide to form peroxynitrite. These molecules may cause cellular damage by reacting with nucleic acids, proteins, and lipids. These reactive substances may target the electron transport chainitself [13], leading to mitochondrial damage and further ROS production. In addition, 6-OHDA, which accumulates in the cytoplasm, promotes the formation of ROS [14]. In an experimental model of PD, increased ROS activates the mitochondrial apoptotic pathway in SN dopaminergic neurons. Neurons initiate a complex network of ROS scavenging to balance this oxidative stress [15,16]. The DJ-1 protein plays a very important role in anti-oxidative stress, anti-apoptosis, and triggering autophagy [17]. Studies have shown that mitochondrial dysfunction is found in Parkinson’s disease patients and DJ-1 knockout mice. The main findings are reduced mitochondrial complex I activity and reduced mitosome membrane potential [18,19]. Under oxidative stress, DJ-1 upregulates SOD-1 expression [20]. Mitochondrial apoptosis is activated by mitogen-activated protein kinases (MAPKs) signaling pathways, which are c-Jun N-terminal kinase (JNK) and P38 mitogen-activated protein kinase (P38). JNK and P38 are essential in mediating SN dopaminergic cell death [16,21]. In addition, apoptosis is an important pathway for PD cell death, and researchers [22] localized apoptosis by detecting the Bcl-2/Bax ratio, mitochondrial cytochrome c release, and Caspase-3 activation. Currently, the use of dopamine agonists, anticholinergic drugs, and enzyme inhibitors is the most widely used treatment strategy for PD. Many diseases are treated using relatively harmless antioxidant molecules found in food. Marine natural products have the potential for the treatment of PD [23]. Marine microalgae-derived peptides are potent antioxidant molecules and are promising candidates for future research [24].

**Figure 1:**
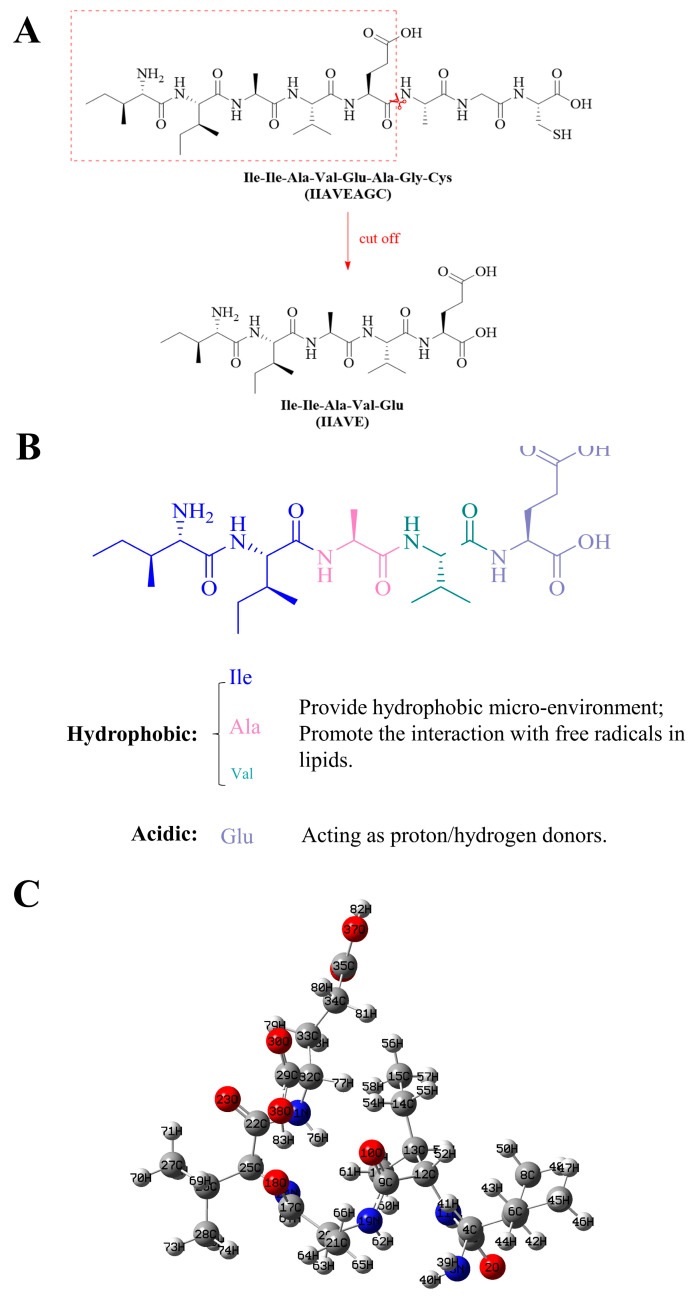
(**A**) Schematic diagram of octapeptide (IIAVEAGC) truncated to pentapeptide (IIAVE); (**B**) structure–antioxidant activity relationship of the IIAVE; (**C**) optimized 3D structure (B3LYP/6-31G(d) in aqueous medium).

Microalgae are the most ubiquitous marine biological resource, which is abundant and easily available [25] and has good biological activities such as antioxidant, anti-inflammatory, anti-cancer, and immune enhancement [26]. *Isochrysis zhanjiangensis* is a microalgae widely distributed in southern China [27]. In previous studies, peptides synthesized from *Isochrysis zhanjiangensis* possessed strong and different biological activities, such as inhibition of tumor angiogenesis [28] and anti-inflammatory effects [29]. In particular, the peptides exemplify powerful antioxidant capacity in the fight against skin photoaging [30] and liver cancer [31]. Typically, bioactive peptides have a molecular weight of less than 6000 Da [32] and contain 2–20 amino acid residues [33]. Peptides of small molecular peptides (500–1500 Da) have higher activity than other peptide sequences [34]. IIAVE (Ile–Ile–Ala–Val–Glu, 543.65 Da) was derived from octapeptide IIAVEAGC (Ile–Ile–Ala–Val–Glu–Ala–Gly–Cys) from *Isochrysis zhanjiangensis* (Figure 1A). The presence of hydrophobic amino acids at the N-terminus of some antioxidant peptides has been reported to increase the antioxidant activity of the peptides [35]. On the other hand, the C-terminus is often occupied by tryptophan, glutamic acid, leucine, isoleucine, methionine, valine, and o-tyrosine [36]. Exposure to glutamic acid at the C-terminus of the IIAVE and hydrophobic N-terminal isoleucine exemplifies its potential antioxidant activity.

The antioxidant activity and active sites of IIAVE in silico and in vitro studies were explored by Lin et al. [37]. However, the structural and chemical characterization aspects of IIAVE are not very clear. In this study, the frontier molecular orbital (FMO), molecular electrostatic potential (MEP), and natural charge (NPA) of IIAVE were calculated using a density-functional theory (DFT) approach. Molecular docking probed the interaction of IIAVE with Bcl-2 and DJ-1 proteins. The present study attempts to elucidate the possibility that the chemical nature of IIAVE interacts with some key proteins. Finally, the antioxidant and neuroprotective effects of IIAVE were further validated in a PD model. Combining the computational and in vivo experimental results, the present study explains the possible mechanisms underlying the neuroprotective effects of IIAVE in alleviating mitochondrial oxidative stress and anti-apoptosis.

## 2. Results

### 2.1. Molecular Docking Analysis

**Figure 2:**
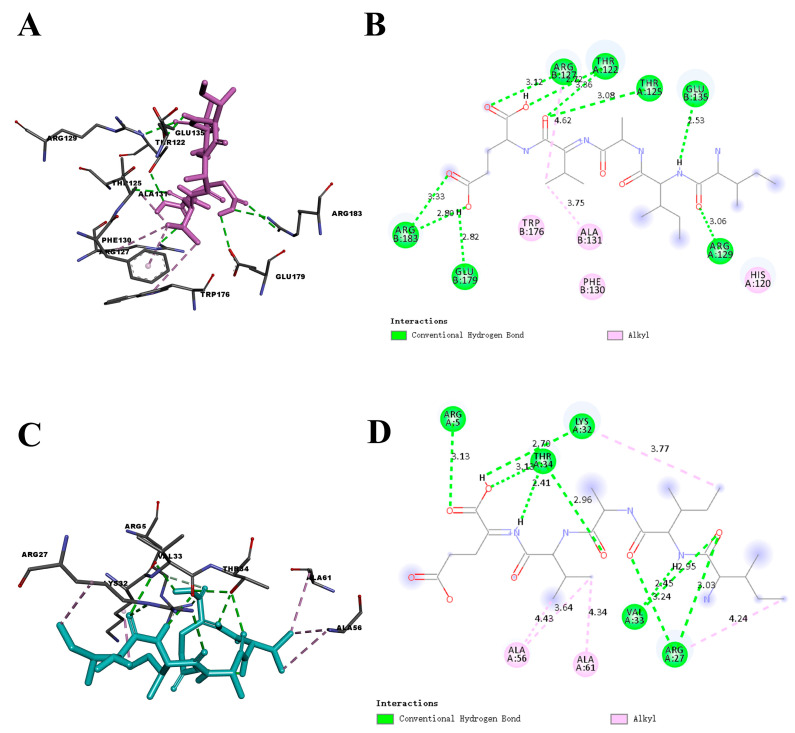
(**A**) The 3D molecular interactions of pentapeptide IIAVE with the Bcl-2 (PDB: 2xa0); (**B**) the 2D molecular interactions of pentapeptide IIAVE with the Bcl-2; (**C**) the 3D molecular interactions of pentapeptide IIAVE with the DJ-1 (PDB: 4rkw); (**D**) the 2D molecular interactions of pentapeptide IIAVE with the DJ-1.

IIAVE was molecularly docked to the key proteins Bcl-2 (PDB: 2xa0) and DJ-1 (PDB: 4rkw), respectively (Figure 2A–D and Table 1). The calculated affinity energies of IIAVE for the best conformations of Bcl-2 and DJ-1 were −6.8 and −5.3 kcal/mol, respectively. A new classification of hydrogen bonding, with binding energies of −2.5 to −14.0 kcal/mol, was reported as “Weak to medium” [38]. The docking results indicated that hydrogen bonding was the main force regulating molecular docking, followed by hydrophobic forces. Thr-125, Thr-122, Arg-129, and Glu-135 of Bcl-2, resulting in 9 hydrogen bonds. A total of 8 hydrogen bonds were generated by the docking of IIAVE with Arg-27, Val-33, Arg-5, Lys-32, and Thr-34 of DJ-1, and most of the hydrogen bonds were located on the Ile, Val, and Glu amino acids of IIAVE. Antioxidant activity is associated with the formation of intramolecular hydrogen bonds. In proteins, hydrogen bonds are mainly formed by the interaction of nitrogen and oxygen atoms in the side chains of amino acids with nitrogen and oxygen atoms in the side or back chains of other amino acids. These hydrogen bonds allow protein molecules to maintain a stable, three-dimensional structure that enables them to carry out their biological functions. Hydrogen bonds are usually between 1.5 and 3.5 Å apart. According to Table 1, the distance between the two key proteins that produce hydrogen bonds is in this range. Based on the affinity energy, hydrogen bond distance, and number of hydrogen bonds, IIAVE binds better to Bcl-2. The results showed that IIAVE docked well with the anti-apoptotic protein Bcl-2 and the anti-oxidative stress protein DJ-1, which laid the foundation for the activity of IIAVE.

Although molecular docking provides valuable insights, it is only a computational prediction and has its limitations. Expression of Bcl-2 and DJ-1 proteins in later cellular experiments will further confirm these findings.

### 2.2. Frontier Molecular Orbital (FMO) and Chemical Reactivity

**Figure 3:**
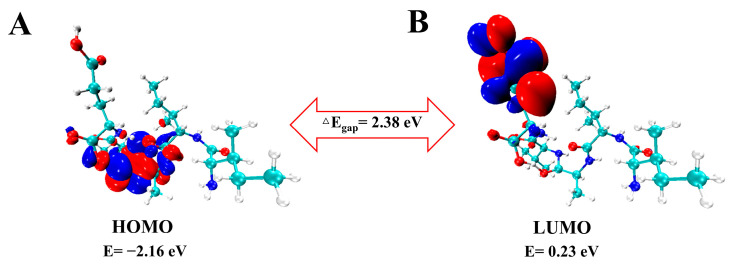
Molecular frontier orbitals of IIAVE. (**A**) HOMO molecular orbital; (**B**) LUMO molecular orbital. Calculated by B3LYP/6-31G(d) in aqueous medium.

The highest occupied molecular orbital (HOMO) and the lowest unoccupied molecular orbital (LUMO) are the relevant molecular orbital energies in a compound (Figure 3A,B). The ability to give electrons is denoted by HOMO, and the ability to gain electrons is denoted by LUMO. Molecules with higher E_HOMO_ values are more likely to act as hydrogen donors, reacting to scavenge free radicals [39]. Molecular docking results show that most of the hydrogen bonds are located on the Ile, Val, and Glu amino acids of IIAVE. After structural optimization, the HOMO orbitals of IIAVE are mainly located on Val and Glu amino acids. Docking results with the molecule similarly showed that Val and Glu amino acids provide protons to form stable hydrogen bonds, providing further data support for the previous molecular docking results. Global reactivity descriptors act as a bridge to understand the relationship between structural stability and global chemical reactivity [40]. Global properties can be acquired by Frontier Molecular Orbital (FMO) energies [41]. HOMO and LUMO are related to ionization potential and electron affinity. The calculated results are shown in Table 2. In order to provide atomic coordinates as auxiliary descriptive information, the optimized atomic coordinates after DFT convergence are shown in Table S1. The chemical descriptors provide the basic chemical properties of IIAVE, which provide potential evidence for its biological activities, such as antioxidant properties.

### 2.3. Molecular Electrostatic Potential (MEP) Analysis

**Figure 4:**
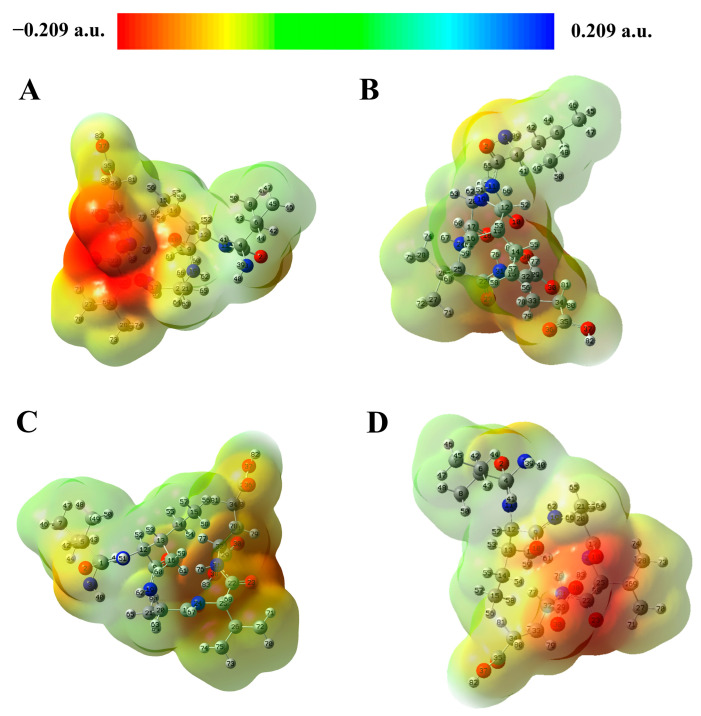
IIAVE molecular electrostatic potential diagram. A perspective view of the electrostatic potential of surface molecules at different angles. Calculated by B3LYP/6-31G(d) in aqueous medium. (**A**). 3D Angle 1; (**B**). 3D Angle 2; (**C**). 3D Angle 3; (**D**). 3D Angle 4.

The molecular electrostatic potential (MEP) shows the properties of a molecule through the total charge distribution on the molecule and helps to predict the molecule’s reactivity to electrophilic and nucleophilic reactions. As shown in Figure 4A–D, the MEP is calculated based on the optimized geometry, with the red regions representing negatively charged regions. These are associated with nucleophilic reactivity. The cyan color represents the neutral region. The blue regions represent positively charged regions related to electrophilic reactivity. Previous reports have mentioned that a hydrogen bond usually exists between negative and positive electrostatic potentials [42]. As can be seen in Figure 4A–D, the regions around O30, O38, O23, O18, and O2 have negative electrostatic potential values but no positive potential regions. These regions have easier access to electrons for hydrogen bonds than the other regions. Negative electrostatic regions indicate the ability to acquire protons, which means that these regions favor hydrogen receptors. In molecular docking results, Bcl-2 is formed with Arg-183, Thr-122, Thr-125, and Arg-129 as hydrogen receptors. Similar results were observed in DJ-1 docking. This result provides further insight into the anti-apoptotic activity and neuroprotective effects of IIAVE.

### 2.4. Natural Population Analysis (NPA)

Atomic charges are generally used to describe the physicochemical properties of molecules (electronic structure, dipole moment, polarization rate, and other molecular properties) [43,44,45,46] and play an important role in molecular structure analysis. The charge observed on atoms is recognized as an important factor in bonding ability and molecular conformation. Pentapeptide unequal electron density redistribution data reveal the presence of highly electronegative elements such as nitrogen and oxygen. Typically, N, O, and F as negative atoms can attract electrons, while the molecular fragment x-H (x being N, O, or F) can release hydrogen atoms to form protons, thus forming new hydrogen bonds. The increase in antioxidant activity is associated with the fact that hydrogen bond donors can form resonance-stabilized free radicals and intramolecular hydrogen bonds [47].

**Figure 5:**
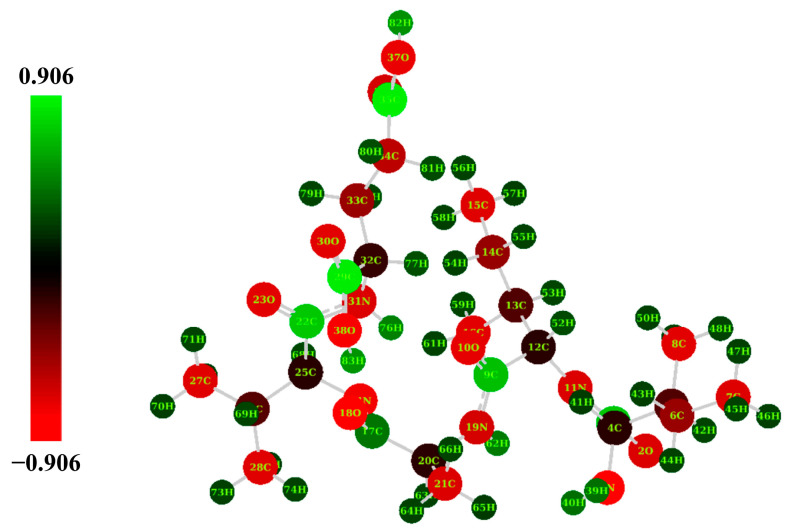
The natural population of IIAVE (e). NPA electron distribution surface of IIAVE (B3LYP/6-31G(d), in aqueous medium).

In the present study, the atomic charges of IIAVE molecules were calculated using NBO analysis at the B3LYP/6-31G(d) level of theory, and the results are shown in Table S2. Figure 5 shows the results for the natural population. The studied charge system is −1. All carbon atoms in IIAVE except C1, C9, C17, C29, and C35 are negatively charged, and all hydrogen atoms are positively charged. N3 (−0.9202 e) and O38 (−0.7480 e) have high electronegativity, while C29 (0.7916 e) and C35 (0.7825 e) have the highest positive charge (Figure 5 and Table S2). The positive carbon (C29 and C35) located on Glu makes the oxygen atoms attached to it more negatively charged and more likely to release protons due to the electron-withdrawal effect. Consistent with the molecular docking results, more negatively charged O38 and N3 atoms act as hydrogen donors and form hydrogen bonds in molecular docking. In conclusion, the NPA results provide fundamental properties of atoms that provide fundamental data for the binding of IIAVE to a number of proteins in living organisms.

### 2.5. Effects of IIAVE and 6-OHDA on the Viability of SH-SY5Y Cells

**Figure 6:**
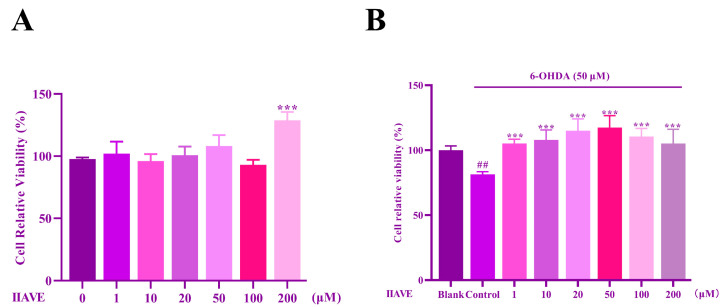
(**A**) Effect of IIAVE (0, 1, 10, 20, 50, 100, and 200 μM) on the viability of SH-SY5Y cells. Compared to the 0 group, *** *p* < 0.001; (**B**) protective effect of IIAVE on 6-OHDA-induced SH-SY5Y. ^#^ Compared to a blank group (SH-SY5Y cells: not treated with IIAVE and 6-OHDA). * Compared to the control group (SH-SY5Y cells: 6-OHDA-induced group without IIAVE treatment). ^##^ *p* < 0.01, *** *p* < 0.001. Data showing mean ± SD (*n* = 3).

To determine the toxicity of IIAVE on SH-SY5Y cells, cell viability was assayed at different concentrations using the CCK-8 kit. Differences were observed between the treated and untreated groups, showing that IIAVE at the highest concentration of 200 µM had no significant toxic effect on the cells (Figure 6A), but had a significant cell proliferation effect. Therefore, IIAVE concentrations of 0, 10, 20, 50, and 100 µM were chosen for subsequent experiments.

6-OHDA is able to be oxidized and decomposed upon entering the cell, generating ROS, further generating oxygen-free radicals, or directly causing mitochondrial dysfunction, leading to DA death [48]. To assess the potential neuroprotective effect of IIAVE, SH-SY5Y cells were pretreated with IIAVE and then exposed to 50 μM of 6-OHDA before cell viability was assessed. Compared to the blank group, incubation of SH-SY5Y cells containing 6-OHDA significantly reduced cell viability (*p* < 0.01, Figure 6B). Compared to the control group, pretreatment with IIAVE at concentrations of 10, 20, 50, and 100 μM significantly restored cell viability (*p* < 0.001, Figure 6B). Therefore, IIAVE has a potential neuroprotective effect against 6-OHDA-induced cytotoxicity in SH-SY5Y cells. This suggests that 50 μM of 6-OHDA-induced modeling of PD disease is feasible.

### 2.6. IIAVE Alleviates Mitochondrial Dysfunction

**Figure 7:**
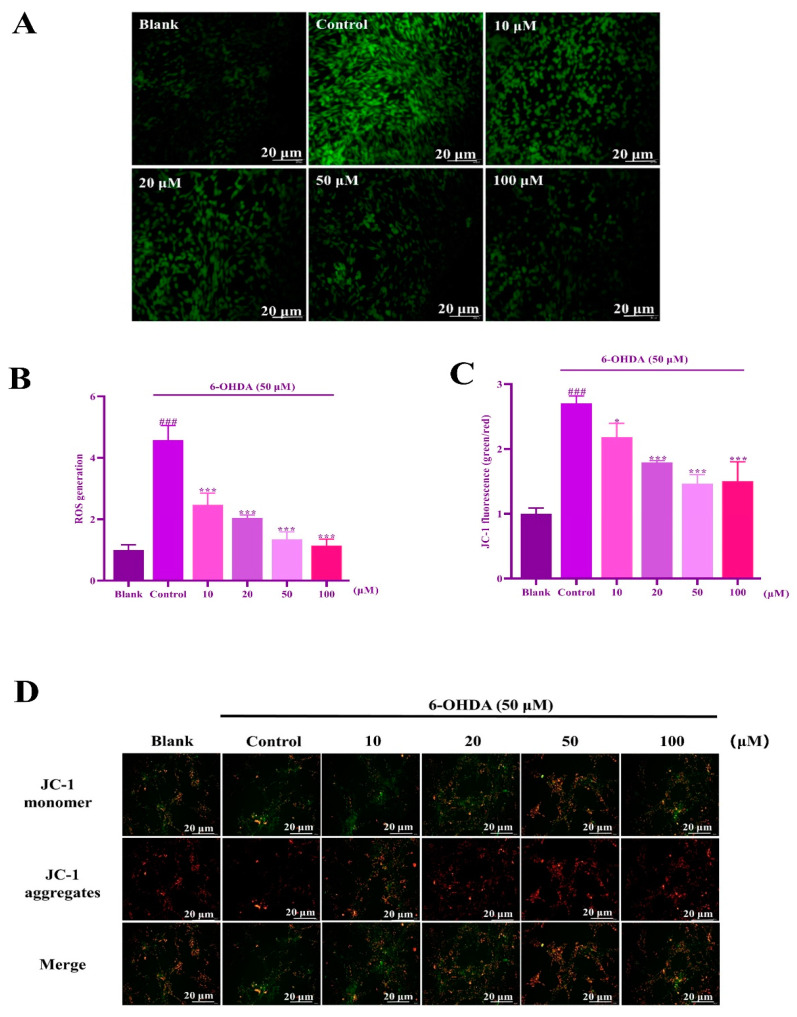
(**A**) Detection of ROS expression by the ROS fluorescent probe; (**B**) assessment of ROS fluorescence intensity using Image J 1.53a; (**C**) assessment of JC-1 fluorescence intensity using Image J; (**D**) detection of mitochondrial membrane potential with JC-1 fluorescent probe. Data are shown as mean ± SD (*n* = 3). ^#^ Compared to a blank group (SH-SY5Y cells: not treated with IIAVE and 6-OHDA). * Compared to the control group (SH-SY5Y cells: 6-OHDA-induced group without IIAVE treatment). ^###^ *p* < 0.001; * *p* < 0.05; *** *p* < 0.001.

To determine intracellular ROS levels and mitochondrial membrane potential, fluorescence intensity was analyzed using a fluorescent probe. Figure 7A,B show a significant increase in cellular ROS levels induced by 6-OHDA in SH-SY5Y cells. Intracellular ROS levels were down-regulated in a concentration-dependent manner by the IIAVE pretreatment group. The JC-1 assay (Figure 7C,D) showed that 6-OHDA damaged the mitochondrial membrane potential, thus promoting early apoptosis in SH-SY5Y cells. However, after IIAVE treatment, the ratio of green fluorescence to red fluorescence of JC-1 decreased, effectively alleviating mitochondrial membrane potential damage and inhibiting apoptosis. The results showed that IIAVE had a good inhibitory effect on 6-OHDA-induced intracellular ROS levels and was able to up-regulate the mitochondrial membrane potential in SH-SY5Y cells.

### 2.7. IIAVE Alleviates Oxidative Stress via the Keap1/Nrf2/SOD-1 Pathway

**Figure 8:**
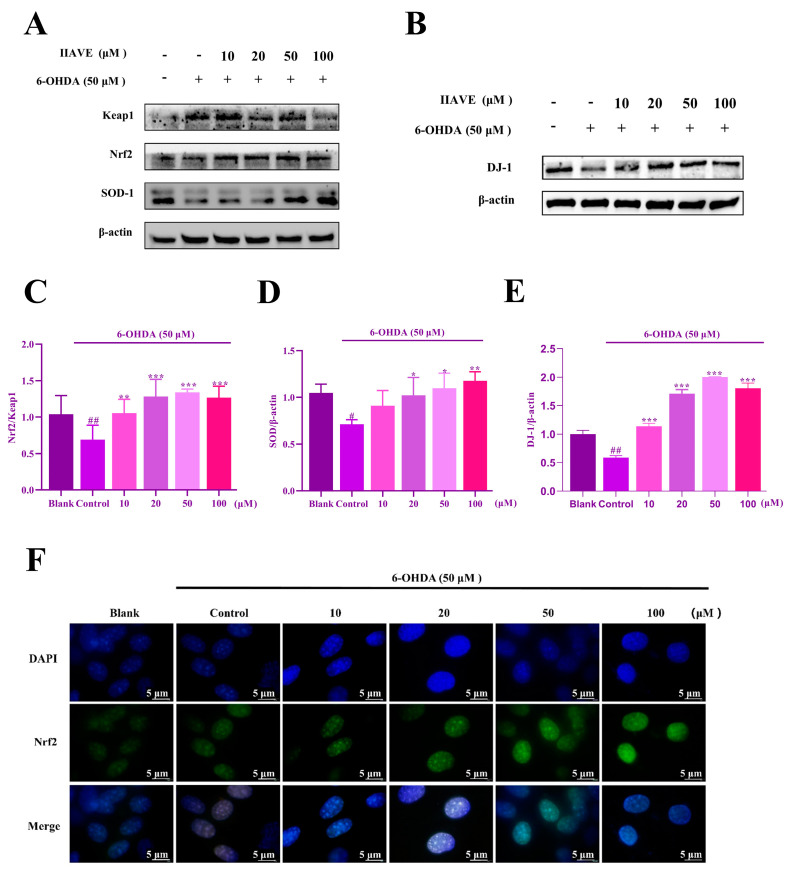
(**A**) Western blotting analysis of the expression levels of Nrf2 pathway-associated protein; (**B**) western blotting analysis of the expression levels of DJ-1 protein; (**C**–**E**) quantification of the expression of these proteins using Image J; (**F**) immunofluorescence analysis of the nuclear expression of Nrf2 protein. Data are shown as mean ± SD (*n* = 3). ^#^ Compared to a blank group (SH-SY5Y cells: not treated with IIAVE and 6-OHDA). * Compared to the control group (SH-SY5Y cells: 6-OHDA-induced group without IIAVE treatment). ^#^ *p* < 0.05; ^##^ *p* < 0.01, * *p* < 0.05; ** *p* < 0.01, *** *p* < 0.001.

Immunofluorescence and Western blot experiments were used to explore the mechanisms of the antioxidant pathway. The results (Figure 8A–E) showed that the expression of Keap1 was increased under 6-OHDA treatment but was reversed after IIAVE pretreatment. The expression of DJ-1 protein was decreased in the 6-OHDA-treated group, and the expression of Nrf2 and SOD-1 proteins followed the same trend as that of DJ-1. In the 6-OHDA-treated group, DJ-1 protein expression was reduced, while Nrf2 and SOD-1 protein expression followed the same trend as that of DJ-1. IIAVE pretreatment increased the expression of DJ-1, Nrf2, and SOD-1 proteins. In addition, Nrf2 immunofluorescence results showed (Figure 8F) that Nrf2 expression was increased in the nuclei of cells in the IIAVE-pretreated group. These results suggest that IIAVE can play an antioxidant role by regulating the Keap1/Nrf2/SOD-1 pathway to alleviate oxidative stress in SH-SY5Y cells through increasing the expression of DJ-1.

### 2.8. IIAVE Inhibits Apoptosis in SH-SY5Y Cells

**Figure 9:**
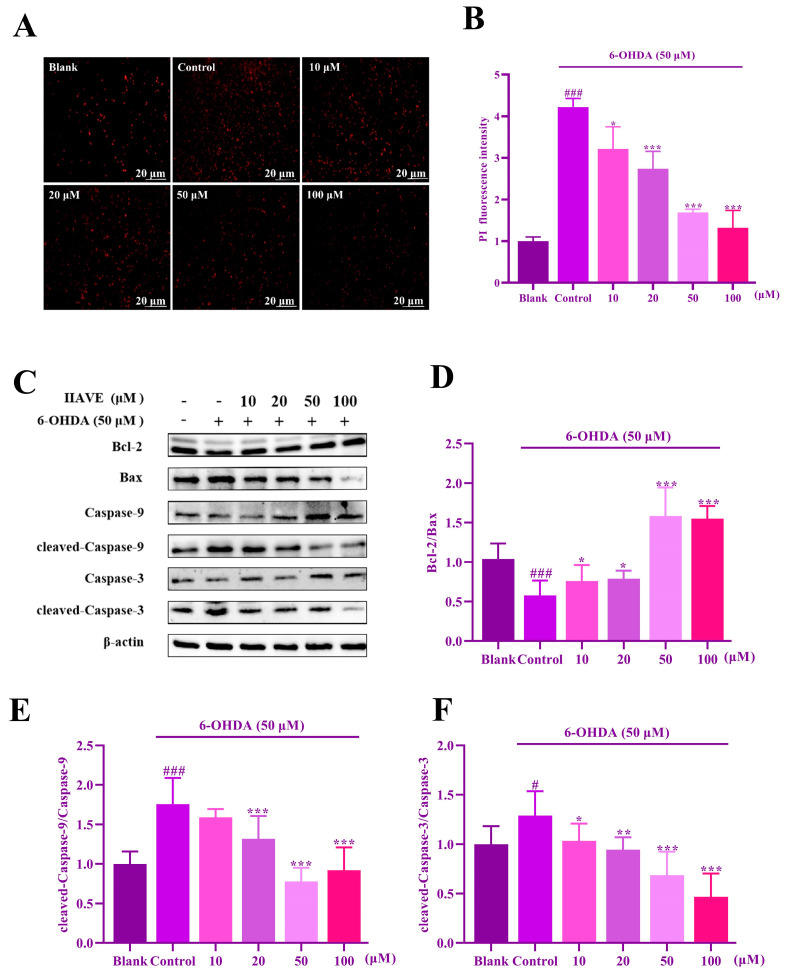
(**A**) Evaluation of the inhibitory effect of IIAVE on 6-OHDA-induced apoptosis using PI staining; (**B**) evaluation of PI fluorescence intensity using Image J; (**C**) western blotting to detect the expression of the apoptosis pathway-associated protein level; (**D**–**F**) quantification of the expression of this protein using Image J. Data are shown as mean ± SD (*n* = 3). ^#^ Compared to a blank group (SH-SY5Y cells: not treated with IIAVE and 6-OHDA). * Compared to the control group (SH-SY5Y cells: 6-OHDA-induced group without IIAVE treatment). ^#^ *p* < 0.05; ^###^ *p* < 0.001; * *p* < 0.05; ** *p* < 0.01, *** *p* < 0.001.

The PI apoptosis assay showed (Figure 9A,B) that, compared to the blank group, the red fluorescence of 6-OHDA was evident with severe apoptosis and damage. After IIAVE treatment, the intensity of the red fluorescence decreased. Furthermore, the expression of apoptosis-related proteins was detected by western blotting, and the results showed (Figure 9C–F) that the pro-apoptotic factors Caspase-3 and Caspase-9 were activated by the 6-OHDA inducer, while IIAVE had a significant inhibitory effect on them. The values of the apoptotic factor Bcl-2/Bax were up-regulated after IIAVE treatment and had a significant effect in the 50 and 100 µM groups. These results suggest that IIAVE can relieve 6-OHDA-induced apoptosis in SH-SY5Y cells.

### 2.9. IIAVE Inhibit JNK and P38 Phosphorylation Elicited by 6-OHDA

**Figure 10:**
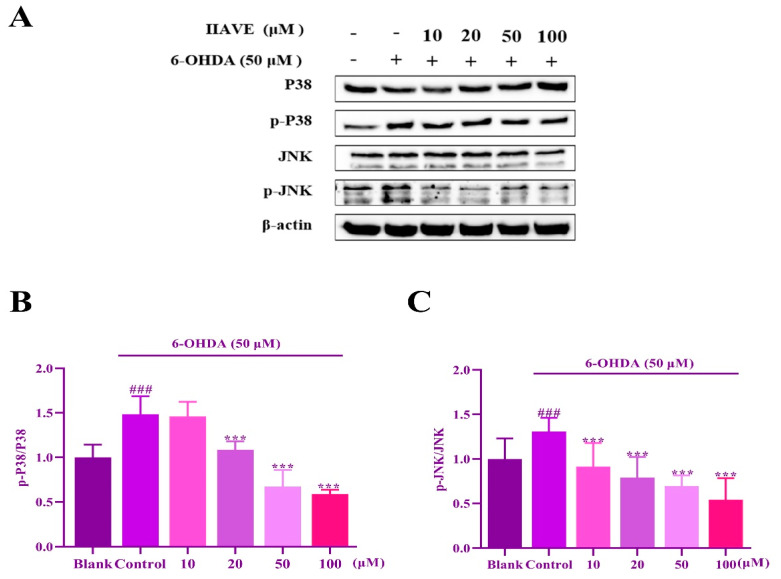
(**A**) The expression of JNK and P38 proteins; (**B**,**C**) quantification of the protein expression by Image J. Data are shown as mean ± SD (*n* = 3). ^#^ Compared to a blank group (SH-SY5Y cells: not treated with IIAVE and 6-OHDA). * Compared to the control group (SH-SY5Y cells: 6-OHDA-induced group without IIAVE treatment). ^###^ *p* < 0.001; *** *p* < 0.001.

The results of protein western blotting (Figure 10A–C) showed that the 6-OHDA-induced group significantly activated the JNK and P38 pathways compared to the blank group, while the phosphorylation of JNK and P38 was reduced in a concentration-dependent manner after the addition of IIAVE treatment. In the low concentrations group, the effect of JNK was more significant than that of P38; in the high concentrations group, the effect of both treatments exceeded that of the blank group, indicating significant pharmacological inhibition. The results indicate that IIAVE inhibits the activation of the JNK and P38 signaling pathways.

### 2.10. Comparison of Antioxidant Properties of Two Peptides

**Figure 11:**
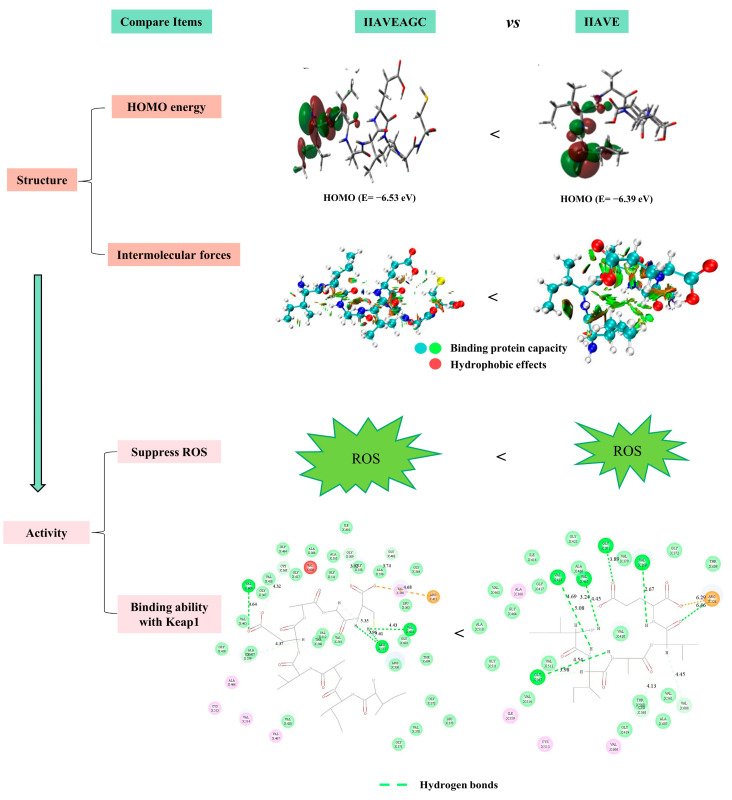
Comparison of octapeptide (IIAVEAGC) with pentapeptide (IIAVE) (Lin et al. 2023) [37].

Lin et al. [37] have compared the structure and activity of two peptides in vitro and in silico (Figure 11). The results showed that the E_HOMO_ of the pentapeptide IIAVE (−6.39 eV) was stronger than that of the octapeptide IIAVEAGC (−6.53 eV). In addition, this IIAVE has the largest hydrophobic effect and the smallest spatial resistance. Electrostatic interactions, hydrogen bonding, and van der Waals forces are some of the main effects of subsequent peptide-protein binding [49]. The blue and green parts reflect a stronger binding capacity. (Red region: strong spatial effects; blue region: electrostatic interactions and hydrogen bonding; green region: van der Waals forces). In vitro experiments show that IIAVE inhibits ROS production stronger than IIAVEAGC. Molecular docking indicates that IIAVE forms stable hydrogen bonds with Keap1. In vitro experiments similarly demonstrated that the antioxidant effect is through the Keap1/Nrf2 pathway.

## 3. Discussion

The antioxidant activity of bioactive peptides has been reported to be mainly due to the presence of hydrophobic amino acids, some aromatic amino acids, and histidine [35,50]. The peptide derivative IIAVE synthesized from *Isochrysis Zhanjiangensis*, which contains hydrophobic amino acids such as Glycine, Alanine, Valine, and Isoleucine, has potent antioxidant activity and has shown antioxidant effects in nerve cells [37]. To maintain the stability of the peptide and exert better antioxidant activity, the peptide was truncated at Glu-5 and Ala-6 of IIAVEAGC to expose more acidic amino acids. In addition, the truncation at Glu-5 and Ala-6 increased the proportion of hydrophobic amino acids to 80% from the previous 62.5%. The exposure of more hydrophobic amino acids at the ends of peptides has an important effect on antioxidant activity. This is because fatty acid radicals are hydrophobic, and they tend to bind first to hydrophobic antioxidant peptides, forming more stable protein structures (Figure 1B). This is also illustrated by the generation of hydrophobic interactions in the molecular docking results.

In order to better understand the chemical and physical properties of the truncated peptides, this study explores the structural properties of IIAVE based on the FMO, NPA, MEP, and global reactivity obtained by density functional theory (DFT). The optimized structure and atom numbering are shown in Figure 5. E_HOMO_ is −2.16 eV, and E_LUMO_ is 0.23 eV (Figure 3). ΔE_gap_ is 2.38 eV. ΔE_gap_ can be used to characterize chemical stability [51]. Typically, when ΔE_gap_ is very small, it has high chemical reactivity. The ionization potential and electron affinity describe the ability to give up and accept electrons. Ionization potential and electron affinity are used to understand the ability to donate and accept electrons. In this study, the ionization potential (I = 2.16 eV) is significantly higher than the electron affinity (A = −0.23 eV). The difference between the FMO analysis and the results of the study by Lin et al. [17] is due to the difference in the charge system used in the calculation. This also suggests that the protonation and deprotonation of peptides under different pH conditions affect biological activity. This does not seem to be a contradiction either; in fact, there are differences in pH inside the human body, such as the pH of organelles [52]. The MEP results showed a highly negative charge in the region near the two O atoms on the carboxyl group of the Glu-5 side chain (O38, O30). The possible reason for this phenomenon is that at physiological pH, the carboxyl group of Glu-5 tends to deprotonate and become negatively charged. Global reactivity descriptors act as a bridge to understand the relationship between structural stability and global chemical reactivity [40]. Global properties can be acquired by Frontier Molecular Orbital (FMO) energies [41]. HOMO and LUMO are related to ionization potential and electron affinity. And chemical hardness and softness are related to ∆E_gap_. A smaller ∆E_gap_ is associated with high chemical softness (*S*), while a higher ∆E_gap_ is associated with chemical hardness (*η*). Chemical hardness and softness can be used as one of the parameters to predict chemical stability [42]. The high η (1.19 eV) and low S (0.40 eV) values also indicate that the structure of the peptide is a soft molecule [43]. In addition, the electrophilic index (ω) and the electrochemical potential (μ) of the polypeptide structure were calculated to be 0.40 eV and −0.97 eV, respectively, and μ and ω are two of the important parameters that provide chemical reactivity. The electronegativity (χ) descriptor is a measure of the ability of an atom or a group of atoms to attract electrons [44]. The electronegativity (χ) of the peptide was calculated to be 0.97 eV. Compounds with low energy gaps (ΔE_gap_) have higher chemical reactivity. Combined with the MEP results, the negative electrostatic potential regions of the pentapeptide are around O30, O38, O23, O18, and O2, implying that these regions have easier access to hydrogen-bonded electrons. This implies that these regions are favorable for hydrogen receptors. Interestingly, the NPA results likewise show that O38 carries a higher negative charge. The presence of highly electronegative O atoms means that the molecular fragment x-H (x for N, O, or F) can release hydrogen atoms to form protons, which in turn form new hydrogen bonds.

Molecular docking of the key proteins Bcl-2 and DJ-1 was performed to further explore the mechanism of cellular activity. The molecular docking results showed that the docking of IIAVE with Bcl-2 and DJ-1 produced 9 and 8 hydrogen bonds, respectively, most of which were on Val and Glu. The MEP results show that the red MEP map (Figure 4A–D) shows that the region around 38O and 30O carries a higher negative charge with a stronger electron-withdrawal-inducing effect, which can lead to an increase in the positive electronegativity of the carbonyl carbon, which is favorable for nucleophilic reactions. The NPA results indicate a lower negative potential near O38, located on Glu, suggesting that O38 acts as a hydrogen acceptor to form hydrogen bonds. The HOMO orbitals are also in the vicinity of Val and Glu, providing the possibility that the reaction occurs. Combining the computational predictions of the two, IIAVE has the potential to alleviate mitochondrial oxidative stress and have anti-apoptotic effects. In cellular experiments, the 6-OHDA-treated group induced apoptosis (Figure 6B and Figure 9A) and mitochondrial dysfunction (Figure 7D). IIAVE pretreatment reversed this effect. Western blot results also showed the same trend in the expression of Bcl-2 (Figure 9C) protein and DJ-1 (Figure 8B). SH-SY5Y cells pretreated with IIAVE significantly increased the Bcl-2/Bax ratio, reducing 6-OHDA-induced apoptosis. This is consistent with other studies [53] that indicated that Acylated ghrelin resisted apoptosis by inhibiting the 6-OHDA-induced increase in the Bax/Bcl-2 ratio. Lai et al. [54] reduced the Bcl-2/Bax ratio in SH-SY5Y cells using 6-OHDA treatment, but treatment with p-Hydroxybenzyl alcohol pretreatment reversed this result. In addition, IIAVE significantly inhibited downstream activation of Caspase-9 and Caspase-3, thereby suppressing apoptosis. This result correlated well with the up-regulation of mitochondrial membrane potential changes by IIAVE pretreatment. In addition, IIAVE inhibited the phosphorylation of JNK and P38 involved in 6-OHDA-induced apoptosis. IIAVE pretreatment significantly inhibited the activation of the JNK and P38-mediated apoptotic pathways, providing further support for the inhibition of 6-OHDA-induced neurotoxicity in SH-SY5Y cells.

To further validate the antioxidant capacity of IIAVE, I conducted in vivo experiments to verify the mechanism by which IIAVE exerts its antioxidant effects. The pharmacological activation of the Nrf2/Keap1 signaling pathway has been shown to alleviate oxidative stress and has been suggested as a potential therapeutic target for PD [55,56,57]. Nrf2 dissociates from Keap1 and translocates into the nucleus, initiating the expression of downstream antioxidant proteins such as SOD-1 [58]. In this study, the fluorescence intensity of Nrf2 was detected by immunofluorescence in vitro experiments. As shown in Figure 8, it can be observed that the fluorescence intensity in the blank and 6-OHDA-stimulated groups was weaker than that in the sample group, implying that IIAVE promoted the expression of Nrf2. In the subsequent Western blot experiments, the expression of Nrf2 promoted the expression of the downstream SOD-1 protein. Studies have shown that IIAVE has good antioxidant activity against H_2_O_2_-induced SH-SY5Y cell damage [37]. E_HOMO_ can be used to explain the molecular electron-donating capacity; the higher the E_HOMO_, the easier the oxidation process. Figure 11 shows that IIAVE has better antioxidant activity compared to the IIAVEAGC peptide. The difference in E_HOMO_ between IIAVE and IIAVEAGC is supposed to be the exposure of the carboxyl group of the Glu-5 side chain. The exposure of the acidic group makes IIAVE more reductive than the uncharged IIAVEAGC. In addition, cellular experiments have also shown that IIAVE can inhibit ROS more effectively [33]. Thus, the mechanism of action for the biological activity of IIAVE in vivo is the scavenging of ROS by binding to Keap1 and releasing Nrf2 to promote the expression of the antioxidant protein SOD-1 [37].

Despite the limitations of computer simulations, cellular experiments further supported these findings. The potential antioxidant and anti-apoptotic activity of IIAVE and the alleviation of mitochondrial stress provide new research strategies for PD therapy. However, the studies are not sufficiently in-depth and, in general, provide new insights into the development of marine active peptides.

## 4. Materials and Methods

### 4.1. Cell Culture and Chemicals

The amino acid sequence of IIAVE was determined as Ile–Ile–Ala–Val–Glu, a derivative peptide from *Isochrysis zhanjiangensis* octapeptide. Based on Gaussian results, Lin et al. [37] truncated peptides at Glu-5 and Ala-6 to retain the finest amino acids required for antioxidant properties. The exposed acidic amino acids contribute to peptide stability. In addition, computer theory investigated hydrophobic interactions, with IIAVE having a greater hydrophobic effect and less spatial resistance compared to IIAVEAGC. Fatty acid radicals are hydrophobic, and they tend to bind first to hydrophobic antioxidant peptides. The position of hydrophobic amino acids affects their antioxidant activity, such as those located at the ends of peptides. The truncated pentapeptide retained the octapeptide antioxidant activity, and the exposed acidic amino acids made the pentapeptide more stable for protein binding. IIAVE was synthesized by Shanghai RoyoBiotech Technology (Shanghai, China) with a purity of ≥98.8%. IIAVE has been shown to have good antioxidant activity in nerve cells [37].

SH-SY5Y, a human neuroblastoma cell line, was purchased from Zhong Qiao Xin Zhou Biotechnology (Shanghai, China) and contained 10% FBS, 1% penicillin, and streptomycin. SH-SY5Y cells were cultured with 5% CO_2_ at 37 °C in DMEM/F12 (1:1) medium, fetal bovine serum (FBS), trypsin-EDTA (0.25%), and phosphate-buffered saline (PBS) from Gibco (Grand Island, NY, USA). The DCFH-DA and JC-1 fluorescent probes were from Beijing Solarbio Science & Technology (Beijing, China). Cell Counting Kit-8 (CCK-8) was purchased from ZETA LIFE (San Francisco, CA, USA). The primary antibodies of P38 (sc-7972), p-P38 (sc-166182), JNK (sc-7345), p-JNK (sc-6254), Caspase-9 (sc-133109), cleaved Caspase-9 (#20750), Caspase-3 (sc-7272), cleaved Caspase-3 (#9661); Keap1 (sc-365626), Nrf2 (sc-81342), SOD-1 (sc-271014), Bax (#5023), Bcl-2 (sc-7382), β-actin (sc-47778) and the secondary antibodies of HRP-labeled goat anti-mouse IgG and HRP-linked anti-rabbit IgG were obtained from Santa Cruz Biotechnology Inc. (Dallas, TX, USA) and Cell Signaling Technology (Danvers, MA, USA); The 6-hydroxydopamine (6-OHDA) was from Shanghai Macklin Biochemical Technology (Shanghai, China); 5X SDS-PAGE protein loading buffer, RIPA lysate, the anti-fluorescent bursting agent were obtained from Shanghai Beyotime Biotechnology (Shanghai, China); All other reagents were used analytically pure.

### 4.2. Research Methods

#### 4.2.1. Cell Viability and Toxicity Assay (CCK-8)

SH-SY5Y cells were plastered all over the culture dish, digested with trypsin, transferred to 48-well plates, and incubated at 90%. An amount of 200 µL of fresh medium was added to contain IIAVE at a range of concentrations (0, 1, 10, 20, 50, 100, and 200 µM) without fetal bovine serum for 24 h, starving overnight. An amount of 20 µL of CCK-8 working solution was added to each well and incubated at 37 °C for 30 min in the dark. Then absorbance values were measured at 450 nm using a BioTek microplate reader (Winooski, VT, USA).

SH-SY5Y cells were cultured in 96-well plates and pretreated with 100 µL of fresh medium containing IIAVE at different concentrations (0, 10, 20, 50, and 100 µM) for 2 h, while a control group was set up with 6-OHDA only. And exposed to 50 µM 6-OHDA maintained at 37 °C for 24 h in an incubator. Then 10 µL of CCK-8 working solution was added to each well and incubated at 37 °C for 30 min in the dark, and absorbance values were measured at 450 nm using a BioTek microplate reader (Winooski, VT, USA).

#### 4.2.2. ROS Generation Detection

SH-SY5Y cells were cultured in 24-well plates and allowed to grow and stabilize. The floating cells were washed with PBS buffer. Various concentrations of IIAVE (0, 10, 20, 50, and 100 µM) were added to the wells and incubated at 37 °C for 2 h, while a control group was set up with 6-OHDA only and exposed to 50 µM 6-OHDA for 24 h. Then concentrations of 10 µM fluorescent probe DCFH-DA were added to incubate at 37 °C for 30 min in the dark, washed twice with PBS buffer, and observed and photographed under an Olympus inverted fluorescent microscope (Tokyo, Japan).

#### 4.2.3. PI Apoptosis Analysis

SH-SY5Y cells were cultured in a complete medium for 24 h. The cells were processed as described in Section 4.2.2. After PBS buffer washing, cells were treated with PI dye at 37 °C for 20 min in the dark. Apoptosis was detected under an inverted fluorescence microscope and photographed.

#### 4.2.4. Mitochondrial Membrane Potential Detection

SH-SY5Y cells were cultured in a complete medium for 24 h. The cells were processed as described in Section 4.2.2 and treated with JC-1 probes at 37 °C for 20 min in the dark. Fluorescence intensity was tested under an inverted fluorescence microscope and photographed.

#### 4.2.5. Immunofluorescence

SH-SY5Y cells were cultured on 24-well plates and allowed to grow on the slides. Cells were processed as described in Section 4.2.2. The old medium was removed, washed three times with pre-cooled PBS buffer to eliminate floating cells, fixed with 4% paraformaldehyde for 30 min, and permeabilized with 0.2% Triton X-100 for 10 min. The cells were blocked with 5% goat serum for 1 h at room temperature and then incubated at 4 °C overnight with Nrf2 primary antibody diluted in 1% BSA and incubated with 488 fluorescent secondary antibodies for 2 h in the dark. Then, the cells were blocked with an anti-fluorescent bursting agent containing DAPI and imaged by inverted fluorescence microscopy.

#### 4.2.6. Western Blotting

SH-SY5Y cells were grown well, digested with trypsin, transferred to 90 mm plates, and treated as described in Section 4.2.2. Cells were fully lysed with RIPA lysis solution containing protease inhibitors, and proteins were stored frozen at −20 °C. Protein samples were centrifuged at high speed, and the supernatant was quantified using a BCA kit. Identical masses of proteins were separated on SDS-PAGE gels, and blots were transferred to nitrocellulose membranes, closed with a TBST shaker containing 7% skimmed milk for 2 h. The TBST was washed three times. The primary antibody was incubated overnight at 4 °C, washed three times with TBST, and incubated with the secondary antibody for 2 h. Eventually, the membrane was developed with an ECL luminescent solution by Tanon Chemiluminescent Instruments (Guangzhou, China).

#### 4.2.7. Molecular Docking

The 3D structures of Bcl-2 (PDB code: 2xa0) and DJ-1 (PDB code: 4rkw) were obtained from the Protein Data Bank (https://www.rcsb.org/ accessed on 23 January 2024), and the IIAVE structure was drawn using ChemDraw 20.0 software. Protein preparation involves removing water molecules, adding hydrogen atoms, and calculating charge. Ligands are processed for energy minimization by Discovery Studio Client v19.1.0. Molecular docking was performed by AutoDockTools Version 1.5.6 [59]. Docking was carried out using semi-flexible docking; the docking parameters were set to 10 times. Visualization was performed using Discovery Studio 2019 software. The lowest binding affinity energy results were chosen for the analysis of docking results.

#### 4.2.8. Quantum Chemistry Research

Two-dimensional structures of molecules were drawn using ChemDraw 20.0. The 3D structures were drawn with Chem3D 20.0 and preliminarily optimized using the molecular force field MM2. The preliminary optimized structures were obtained by Gaussian calculations. The Gaussian 09 Windows version was used for structure optimization, frequency calculation, FMO, MEP and NPA analysis. All calculations were performed at the B3LYP/6-31G(d) level and corrected for dispersion using GD3BJ with a peptide charge of −1 in aqueous medium. The PCM continuum solvation model was used in the calculation. Visualization of results using GaussView 5.0.8. HOMO and LUMO track drawings using Multiwfn 3.8 dev [60] and VMD [61]. The electronic features were analyzed through the highest occupied molecular orbital energy (E_HOMO_), the lowest unoccupied molecular orbital energy (E_LUMO_), the gap energy (ΔE_gap_), the dipole moment, the ionization potential (I), the electron affinity (A), the electronegativity (χ), the electrochemical potential (µ), the electrophilicity index (ω), global hardness (*η*), and global softness (S). (1)–(8), as shown in the literature [62,63]:

I ≈ −E_HOMO_ (1)

A ≈ −E_LUMO_ (2)

ΔE_gap_ = E_LUMO_ − E_HOMO_ (3)

(4) $$\chi =\frac{I+A}{2}$$

(5) $$\mu =-\frac{I+A}{2}$$

(6) $$\eta =\frac{I-A}{2}$$

(7) $$\omega =\frac{\mu^{2}}{2\eta }$$

(8) $$S=\frac{\chi^{2}}{2\eta }$$

#### 4.2.9. Statistical Analysis

All results are expressed as mean ± S.D (*n* = 3). At least three independent experiments were performed, and statistical analysis was performed using the one-way ANOVA (analysis of variance) test. Compared to the blank control group, *^###^* indicates *p* < 0.001, a highly significant difference, *^##^* indicates *p* < 0.01, a significant difference; *^#^* indicates *p* < 0.05, a significant difference; compared to the control group, *** indicates *p* < 0.001, a highly significant difference; ** indicates *p* < 0.01, a significant difference; * indicates *p* < 0.05, a significant difference.

## 5. Conclusions

**Figure 12:**
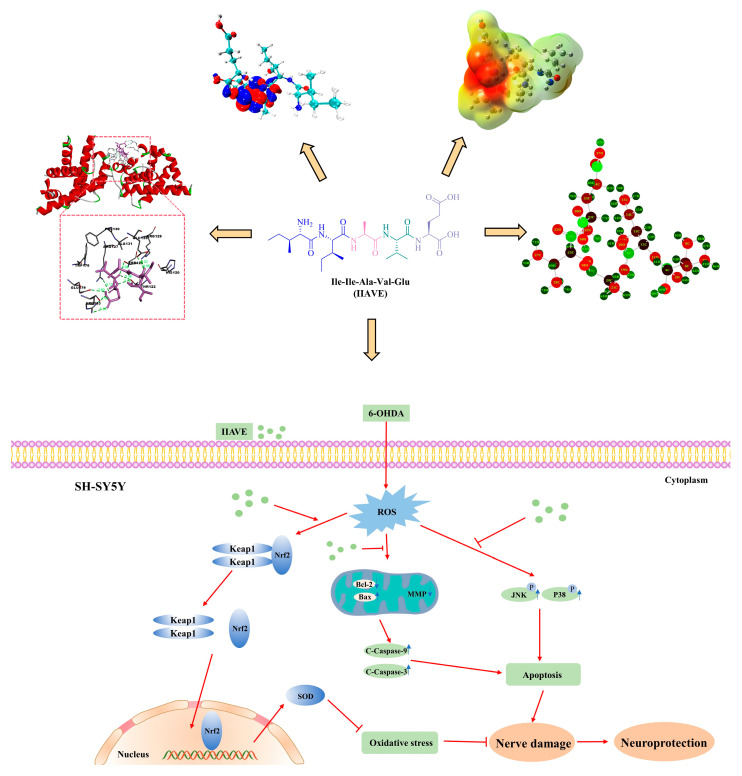
Properties of IIAVE chemical structure and main signal pathway inhibiting oxidative stress in SH-SY5Y cells.

In conclusion, the present study provides some new insights to investigate the chemical properties of pentapeptide IIAVE and the mechanisms of anti-oxidative stress and anti-apoptosis in PD disease models. Molecular docking and quantum chemical calculations elucidated the possibility of IIAVE binding to Bcl-2 and DJ-1 proteins. The results of in vitro experiments showed that IIAVE could restore mitochondrial membrane potential by inhibiting ROS production, promoting the expression of the Keap1/Nrf2/SOD-1 cell signaling pathway, reducing the Bcl-2/Bax ratio, and thus inhibiting the activation of Caspase-9/Caspase-3. This suggests that IIAVE has potential neuroprotective effects (Figure 12). This study informs the study of small-molecule-derived peptides and microalgal molecular mechanisms, providing fundamental insights into food-based peptide therapeutic strategies for PD. This provides additional data support for the future development of marine bioactive peptides.

## Figures and Tables

**Table 1:** IIAVE contacts with Bcl-2 and DJ-1.

Protein	Residue	Interaction Type	Distance (Å)	Protein	Residue	Interaction Type	Distance (Å)
Bcl-2	Arg-183	H-Bond	2.80	DJ-1	Arg-27	H-Bond	3.03
	Arg-183	H-Bond	3.33		Val-33	H-Bond	2.95
	Glu-179	H-Bond	2.82		Arg-27	H-Bond	3.24
	Arg-127	H-Bond	3.12		Val-33	H-Bond	2.45
	Thr-125	H-Bond	3.08		Lys-32	H-Bond	2.70
	Thr-122	H-Bond	2.72		Arg-5	H-Bond	3.13
	Thr-122	H-Bond	3.36		Thr-34	H-Bond	2.41
	Arg-129	H-Bond	3.06		Thr-34	H-Bond	2.96
	Glu-135	H-Bond	2.53		Ala-61	Hydrophobic	4.34
	Arg-127	Hydrophobic	4.62		Ala-56	Hydrophobic	4.43
	Ala-131	Hydrophobic	3.75		Ala-56	Hydrophobic	3.64
					Lys-32	Hydrophobic	3.77
					Arg-27	Hydrophobic	4.24

**Table 2:** Global reactivity descriptors indicate the reactive behavior of pentapeptide IIAVE.

Parameter (eV)	Calculated Result
E_HOMO_	−2.16
E_LUMO_	0.23
ΔE_gap_	2.38
I	2.16
A	−0.23
*χ*	0.97
*µ*	−0.97
*η*	1.19
*ω*	0.40
S	0.40

## Data Availability

The authors confirmed that the data supporting the findings of this study are available within this article.

## Supplementary Materials

The following supporting information can be downloaded at: https://www.mdpi.com/article/10.3390/molecules29030692/s1, Table S1. Optimized atomic coordinates; Table S2. The natural population of the IIAVE.
