# Supplementary material for: Neuroprotection of Truncated Peptide IIAVE from Isochrysis zhanjiangensis: Quantum Chemical, Molecular Docking, and Bioactivity Studies
Source: Molecules. 2024 Feb 2;29(3):692. doi: 10.3390/molecules29030692 (PMC10856281; doi:10.3390/molecules29030692)
Supplement: Supplementary file 1 [file molecules-29-00692-s001.zip › molecules-2733273-supplementary.pdf]

Table S1. Optimized atomic coordinates.

		Coordinates (Å)					Coordinates (Å)		
**NO.**	**Atom**	**X**	**Y**	**Z**	**NO.**	**Atom**	**X**	**Y**	**Z**
**1**	C	-4.398246	-0.43367	1.279267	**43**	H	-4.883465	1.225117	-2.548198
**2**	O	-5.343018	-0.838839	1.955921	**44**	H	-6.178445	0.038354	-2.402376
**3**	N	-4.99506	-1.528542	-0.796558	**45**	H	-7.102654	2.031045	-3.461408
**4**	C	-4.532332	-0.244359	-0.236505	**46**	H	-7.805393	1.90334	-1.840646
**5**	C	-5.552798	0.884138	-0.518941	**47**	H	-6.523536	3.081007	-2.164059
**6**	C	-5.827683	1.006621	-2.027724	**48**	H	-5.81787	2.99522	-0.02493
**7**	C	-6.87096	2.068028	-2.391243	**49**	H	-4.846151	2.114751	1.151384
**8**	C	-5.064719	2.209842	0.082205	**50**	H	-4.152063	2.554392	-0.420557
**9**	C	-1.302843	-0.435145	0.229678	**51**	H	-3.206272	-0.24514	2.871094
**10**	O	-0.576996	0.088771	-0.630026	**52**	H	-2.321982	1.355555	0.695068
**11**	N	-3.192493	-0.158175	1.860788	**53**	H	-1.711755	1.468678	3.106715
**12**	C	-2.016131	0.468458	1.256857	**54**	H	0.621851	1.478605	1.153013
**13**	C	-1.063109	0.943575	2.389911	**55**	H	-0.587446	2.738121	1.293527
**14**	C	-0.046108	1.970411	1.861384	**56**	H	1.449123	3.402207	2.515455
**15**	C	0.786651	2.647093	2.952831	**57**	H	0.147233	3.142759	3.693893
**16**	C	-0.389821	-0.23323	3.111327	**58**	H	1.419773	1.928519	3.483882
**17**	C	0.663626	-2.757762	-0.55509	**59**	H	0.154317	0.115561	3.99335
**18**	O	1.282612	-2.375533	-1.649036	**60**	H	-1.121799	-0.973138	3.454973
**19**	N	-1.519527	-1.754234	0.337144	**61**	H	0.315284	-0.748267	2.448871
**20**	C	-0.846977	-2.837155	-0.465966	**62**	H	-2.091858	-2.046566	1.119323
**21**	C	-1.483279	-2.915181	-1.852819	**63**	H	-1.124201	-3.732557	0.109774
**22**	C	3.198844	-1.11647	0.003825	**64**	H	-1.060128	-3.751927	-2.41585
**23**	O	4.318568	-0.980623	-0.500648	**65**	H	-2.566364	-3.051904	-1.775132
**24**	N	1.280205	-2.442832	0.717181	**66**	H	-1.27966	-1.995485	-2.405468
**25**	C	2.74153	-2.385588	0.75034	**67**	H	0.918581	-3.032217	1.462102
**26**	C	3.504796	-3.652453	0.273144	**68**	H	3.00444	-2.224737	1.80681
**27**	C	4.936521	-3.670155	0.825559	**69**	H	3.542664	-3.618042	-0.820622
**28**	C	2.762491	-4.927756	0.689141	**70**	H	5.480439	-4.552511	0.466446
**29**	C	2.737468	0.681689	-2.309916	**71**	H	5.483601	-2.775319	0.523744
**30**	O	3.422618	1.426663	-3.00374	**72**	H	4.923376	-3.715165	1.923478
**31**	N	2.270929	-0.128371	-0.007518	**73**	H	3.327623	-5.814602	0.378867
**32**	C	2.393142	1.042949	-0.854626	**74**	H	1.770649	-4.971341	0.228278
**33**	C	3.314383	2.1045	-0.242403	**75**	H	2.641517	-4.978611	1.779906
**34**	C	3.065091	3.50894	-0.792215	**76**	H	1.326455	-0.429075	0.206584
**35**	C	3.635205	4.590897	0.09274	**77**	H	1.376769	1.450183	-0.904697
**36**	O	3.861814	4.494519	1.284322	**78**	H	3.127279	2.112529	0.834857
**37**	O	3.846885	5.738794	-0.588997	**79**	H	4.359306	1.815971	-0.388039
**38**	O	2.184377	-0.403379	-2.80872	**80**	H	3.449756	3.616287	-1.807508
**39**	H	-4.836638	-1.513683	-1.80238	**81**	H	1.983475	3.703892	-0.85056
**40**	H	-4.395537	-2.276007	-0.447866	**82**	H	4.170748	6.398038	0.055047

		Coordinates (Å)					Coordinates (Å)		
NO.	Atom	X	Y	Z	NO.	Atom	X	Y	Z
41	H	-3.56988	0.064164	-0.665229	83	H	1.722828	-1.105151	-2.144821
42	H	-6.485694	0.589956	-0.020546	-	-	-	-	-

Table S2. The natural population of the IIAVE.

NO.	Atom	Charge	NO.	Atom	Charge	NO.	Atom	Charge
1	C	0.6998	29	C	0.7916	57	H	0.2240
2	O	-0.6557	30	O	-0.6629	58	H	0.2248
3	N	-0.9202	31	N	-0.6742	59	H	0.2460
4	C	-0.1432	32	C	-0.1791	60	H	0.2243
5	C	-0.2582	33	C	-0.4638	61	H	0.2178
6	C	-0.4623	34	C	-0.5630	62	H	0.4316
7	C	-0.6807	35	C	0.7825	63	H	0.2536
8	C	-0.6916	36	O	-0.6809	64	H	0.2408
9	C	0.6036	37	O	-0.7266	65	H	0.2252
10	O	-0.6956	38	O	-0.7480	66	H	0.2378
11	N	-0.6692	39	H	0.3939	67	H	0.4250
12	C	-0.1363	40	H	0.3875	68	H	0.2604
13	C	-0.2578	41	H	0.2317	69	H	0.2496
14	C	-0.4622	42	H	0.2459	70	H	0.2279
15	C	-0.6831	43	H	0.2310	71	H	0.2417
16	C	-0.6785	44	H	0.2352	72	H	0.2220
17	C	0.5797	45	H	0.2373	73	H	0.2340
18	O	-0.7361	46	H	0.2285	74	H	0.2358
19	N	-0.6851	47	H	0.2288	75	H	0.2229
20	C	-0.1383	48	H	0.2422	76	H	0.4559
21	C	-0.6798	49	H	0.2371	77	H	0.2605
22	C	0.6720	50	H	0.2267	78	H	0.2478
23	O	-0.6914	51	H	0.4275	79	H	0.2462
24	N	-0.6797	52	H	0.2545	80	H	0.2556
25	C	-0.1434	53	H	0.2461	81	H	0.2416
26	C	-0.2643	54	H	0.2423	82	H	0.4998
27	C	-0.6801	55	H	0.2344	83	H	0.5192
28	C	-0.6800	56	H	0.2397	-	-	-
